# We need to talk—how muscle stem cells communicate

**DOI:** 10.3389/fcell.2024.1378548

**Published:** 2024-07-10

**Authors:** Karolina Majchrzak, Erik Hentschel, Katja Hönzke, Christiane Geithe, Julia von Maltzahn

**Affiliations:** ^1^ Faculty of Health Sciences Brandenburg, Brandenburg University of Technology Cottbus–Senftenberg, Senftenberg, Germany; ^2^ Department of Infectious Diseases and Respiratory Medicine, Charité-Universitätsmedizin Berlin, Corporate Member of Freie Universität Berlin and Humboldt Universität zu Berlin, Berlin, Germany; ^3^ Leibniz Institute on Aging, Fritz Lipmann Institute, Jena, Germany; ^4^ Faculty for Environment and Natural Sciences, Brandenburg University of Technology Cottbus—Senftenberg, Senftenberg, Germany

**Keywords:** muscle stem cell, satellite cell, skeletal muscle, regeneration, niche, receptor, aging, rhabdomyosarcoma

## Abstract

Skeletal muscle is one of the tissues with the highest ability to regenerate, a finely controlled process which is critically depending on muscle stem cells. Muscle stem cell functionality depends on intrinsic signaling pathways and interaction with their immediate niche. Upon injury quiescent muscle stem cells get activated, proliferate and fuse to form new myofibers, a process involving the interaction of multiple cell types in regenerating skeletal muscle. Receptors in muscle stem cells receive the respective signals through direct cell-cell interaction, signaling via secreted factors or cell-matrix interactions thereby regulating responses of muscle stem cells to external stimuli. Here, we discuss how muscle stem cells interact with their immediate niche focusing on how this controls their quiescence, activation and self-renewal and how these processes are altered in age and disease.

## Introduction

Skeletal muscle fulfills a variety of functions in the body and makes up over 40% of the human body weight (Frontera and Ochala, 2015). The essential functions of skeletal muscle include respiration, locomotion, body posture, thermogenesis, carbohydrate and amino acid storage as well as glucose and energy metabolism of the body (Jensen et al., 2011; Rowland et al., 2015; Sharma et al., 2019). Moreover, skeletal muscle tissue is also responsible for the secretion of messenger molecules to facilitate communication with other tissues (Pedersen and Febbraio, 2012). Loss of muscle mass and functionality, e.g., due to hormonal changes, malnutrition, aging or disease, can have a prominent impact on the quality of life and general health (Larsson et al., 2019).

### The components of skeletal muscle

**FIGURE 1:**
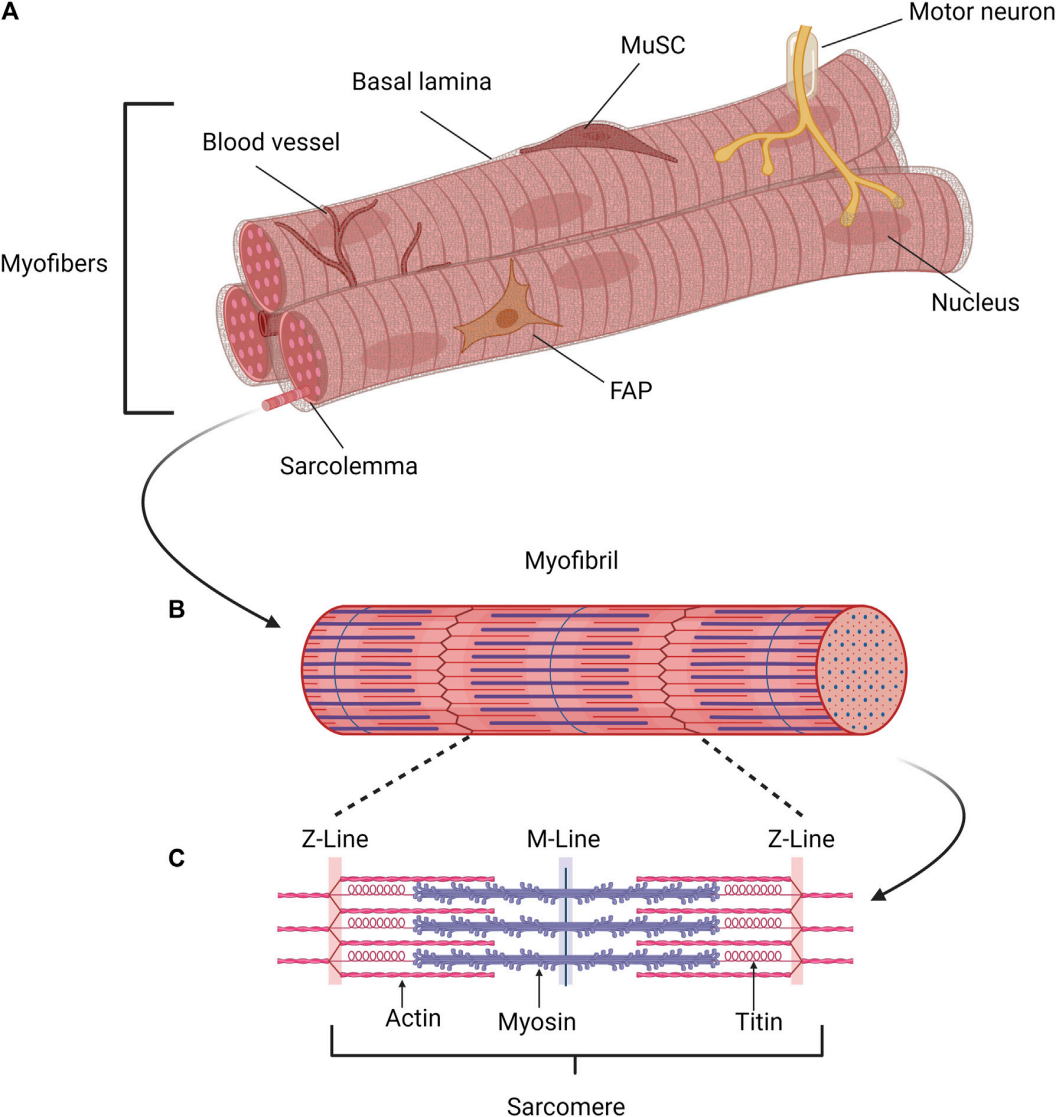
Schematic of the structure of skeletal muscle. **(A)** Myofibers, connective tissue, blood vessels, muscle stem cells (MuSCs), and various support cell types, such as fibrogenic adipogenic progenitor cells (FAPs), are found in skeletal muscle. Motor neurons innervate the multinucleated myofibers, enabling coordinated movement. The postmitotic myofiber is the primary cell type in skeletal muscle, responsible for force production and muscular contraction. **(B)** Each myofiber contains several parallel myofibrils, composed of repeating contractile units, the sarcomeres. **(C)** Muscle contraction is mediated by the sarcomere, the smallest contractile unit formed by overlapping elastic, thick, and thin filaments. Created with BioRender.com.

For fulfilling its essential functions skeletal muscle consists of a multitude of cell types including myofibers, blood vessels, muscle stem cells as well as different support cells such as fibrogenic adipogenic progenitor cells (FAPs) (Figure 1A). Furthermore, the multinucleated myofibers are innervated by motor neurons, which facilitate coordinated movements (Heckman and Enoka, 2012). However, postmitotic myofibers make up the largest portion of cells in skeletal muscle (Figure 1A) containing several myofibrils (Figure 1B) and are allowing muscle contraction and force generation (Dave et al., 2024). Contraction of skeletal muscle depends on its smallest contractile unit, the sarcomere (Figure 1C), consisting of thin and thick myofilaments. The thin myofilaments are composed of two filamentous actin chains (α-actin) which are anchored at the Z-discs (Cooper and September, 2008; Frontera and Ochala, 2015; Wang et al., 2021), while the thick myofilaments are formed by several hundred myosin motor proteins, which slide on top of the thin myofilaments and thereby accomplish contraction of skeletal muscle. A third myofilament, titin, is required for regulating force generation, sarcomere organization and mechanosensing (Linke and Kruger, 2010; Nishikawa et al., 2020).

### Different cell types in adult skeletal muscle

Skeletal muscle requires a multitude of cell types for full functionality and to allow proper regeneration. While contraction is carried out by myofibers, muscle stem cells (MuSCs) and different kind of support cells such as FAPs (fibrogenic adipogenic progenitor cells) are required for its regeneration. A fine network of blood vessels provide myofibers with oxygen and nutrients, while motor neurons are required for coordinated contraction of myofibers and thereby coordinated movements. Blood vessels provide oxygen and nutrients and motor neurons are required for coordinated movement of skeletal muscle. Interaction of the different cell types in skeletal muscle–either through direct cell-cell contact or via paracrine signaling–is required for homeostasis and full functionality of skeletal muscle and is a prerequisite for regeneration of skeletal muscle.

Myofibers make up the largest proportion of skeletal muscle, they contain the sarcoplasmic reticulum and the mitochondrial network within the inter-myofilament space which is providing storage, release and reuptake of calcium after activation as well as ATP for muscle activity (Hargreaves and Spriet, 2020; Rossi et al., 2022). Of note, the myonuclei are evenly distributed in myofibers resulting in the control of transcriptional activity in the surrounding area of the cytoplasm which is termed the myonuclear domain (Qaisar and Larsson, 2014). However, at neuromuscular junctions (NMJ) an accumulation of nuclei occurs (Bruusgaard et al., 2003).

Each myofiber is surrounded by a basal lamina consisting of different collagens and laminins among other proteins. The endomysium, a fibrillar connective tissue surrounding each myofiber, forms a continuous three-dimensional network and provides a connection between adjacent myofibers (Sanes, 2003; Purslow, 2020). MuSCs are located underneath the basal lamina next to the myofibers (Figure 1A). Regeneration of skeletal muscle is crucially dependent on those adult stem cells which are also termed satellite cells (Lepper et al., 2011; Murphy et al., 2011; Sambasivan et al., 2011). In addition to their role in regeneration of skeletal muscle, MuSCs are contributing to adaptation of skeletal muscle to physiological demands such as training and growth. Under resting conditions, MuSCs are mitotically quiescent and are characterized by the expression of paired box protein 7 (Pax7), sprouty-1, and calcitonin receptor (CalcR) among others (Fuchs and Blau, 2020; von Maltzahn, 2021; Yamaguchi et al., 2015; Yin et al., 2013). Several myofibers with their adjacent MuSCs are grouped into muscle fascicles or myofiber bundles, which are surrounded by a second connective tissue termed the perimysium. The complete muscle is composed of a multitude of muscle fascicles, surrounded by a thick layer of connective tissue, the epimysium, which is extending from the tendons (Zhang W. et al., 2021). This connective tissue is maintained by residual fibroblasts in skeletal muscle (Purslow, 2020). It provides the connection of the myofiber bundles to the tendons while the vasculature supplies the individual myofibers with nutrients, oxygen or signal molecules and removes waste products. The vasculature consists of endothelial cells, smooth muscle cells and connective tissue which are embedded as small capillaries in the endomysium (Pittman, 2000; Korthuis, 2011). However, during regeneration of skeletal muscle new myofibers are formed along with the different layers of connective tissue. Especially during regeneration tissue monocytes and differentiated macrophages play fundamental roles including the removal of cell debris. Differentiated macrophages arise either from residential monocytes within the muscle tissue or are entering skeletal muscle via the bloodstream (Pillon et al., 2013).

To allow proper control of muscle contraction, motor neurons are in close contact with individual myofibers at the NMJs (Figure 1A). Typically, only one NMJ is connected to one myofiber (Rodriguez Cruz et al., 2020). These chemical synapses are located between a myofiber and a motor neuron, allow the signal transmission from the neuron to the myofiber and control the induction of contraction of individual myofibers (Ang et al., 2022). The neurotransmitter acetylcholine (ACh) binds to acetylcholine receptors (AChRs) in myofibers after release by the motor neuron. AChR subunits undergo a conformational change resulting in the influx of positively charged ions changing the membrane potential thereby triggering an endplate potential resulting in local depolarization. The generated action potential is spreading from the endplate finally resulting in muscle contraction (Sanes and Lichtman, 1999; Rodriguez Cruz et al., 2020). Performance of skeletal muscle declines if innervation and signal transmission via NMJs are impaired, a condition occurring for instance during aging and in neuromuscular pathologies such as spinal muscular atrophy. This emphasizes the need for proper innervation of skeletal muscle (Tintignac et al., 2015). However, loss of innervation also affects regeneration of skeletal muscle (Jejurikar et al., 2002; Wong et al., 2021; Henze et al., 2024). Of note, also MuSCs actively participate in regeneration of the NMJ underscoring the importance of proper crosstalk between NMJs and MuSCs (Liu et al., 2015; Liu et al., 2017).

The myotendinous junction (MTJ) regulates force transmission between myofibers and tendons (Charvet et al., 2012). MTJs are responsible for transmitting the force which is generated by the muscle to the collagen fibers of the adjacent tendon (Ciena et al., 2010). Recent studies have provided insights into the development and regeneration of muscles and MTJs. These findings indicate that even in case of severely damaged MTJs they can still undergo regeneration, a process which occurs simultaneously with regeneration of muscle tissue and allows full functionality of skeletal muscle after completion of the regeneration process (Yamamoto et al., 2022).

### Regeneration of skeletal muscle

**FIGURE 2:**
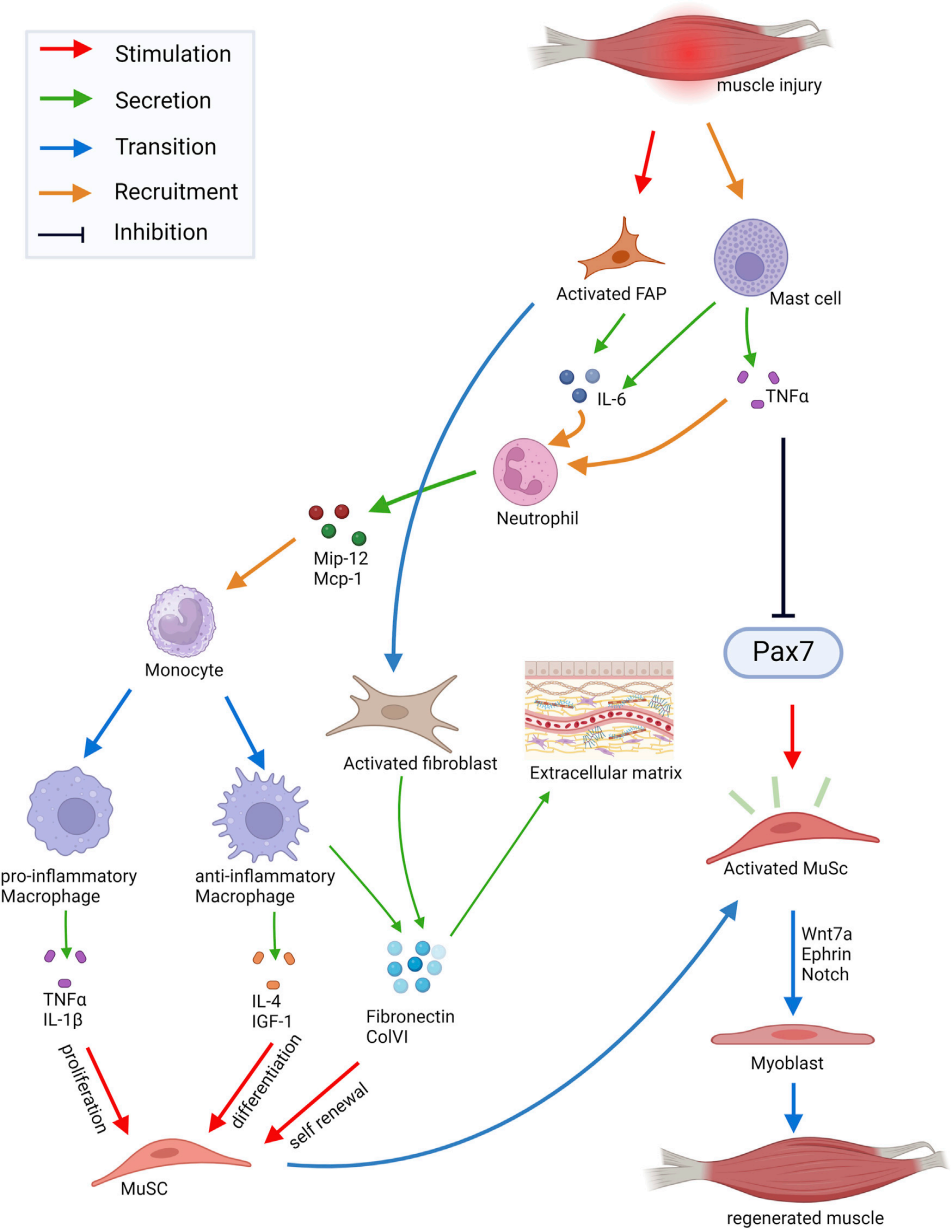
Schematic of cell-cell interactions in skeletal muscle during regeneration. Injury of skeletal muscle triggers mast cells secreting TNF-α and IL-6. This leads to the rapid attraction of granulocytes mainly consisting of neutrophils. Secreted chemokines (Mip-1α, Mcp-1) recruit monocytes which then start to differentiate into pro- and anti-inflammatory macrophages. The pro-inflammatory macrophages secrete TNF-α and IL-1β inducing proliferation of MuSC (muscle stem cells), whereas factors secreted by anti-inflammatory macrophages, such as IL-4 or IGF-1, stimulate myogenic differentiation. Moreover, ECM proteins secreted by anti-inflammatory macrophages, such as Fibronectin and ColVI, promote self-renewal of MuSCs. Upon injury, MuSCs leave the quiescent state and enter the cell cycle. Activated MuSCs can migrate to the site of injury and fuse with the damaged myofibers, which is controlled by Ephrins and Wnt7a signaling. Abbreviations: FAP, fibrogenic adipogenic progenitor cells; IL, Interleukin; TNFɑ, Tumor necrosis factor α; Mip-12, Macrophage Inflammatory Protein 12; Mcp-1, Monocyte Chemotactic Protein 1; IGF-1, Insulin Growth-Like Factor 1; ColVI, Collagen type VI. Created with BioRender.com.

Skeletal muscle is one of the tissues with the highest ability to regenerate after injury, a process which involves different cell types residing in skeletal muscle and requires a proper cross talk among them (Bentzinger et al., 2013a) (Figure 2). The fine balance between different signaling pathways and proper timing of cellular processes are a prerequisite for effective regeneration of skeletal muscle. Regeneration of skeletal muscle can be divided in the following phases: the phase of degeneration, the inflammatory phase, the regeneration phase and the maturation/remodeling phase followed by functional recovery (Schmidt et al., 2019; Forcina et al., 2020).

Injury of skeletal muscle triggers a precisely orchestrated inflammatory process (Figure 2). Damage-activated mast cells secrete Tumor Necrosis Factor α (TNF-α), histamine and Tryptase and then initiate the synthesis of cytokines like IL-6 (Gibbs et al., 2001). This leads to the rapid attraction of circulating granulocytes mainly consisting of neutrophils which promote the proinflammatory environment required for the clearance of cellular debris (Tidball, 1995). Neutrophils then secrete the chemokines Mip-12, Mcp-1 among others leading to the recruitment of monocytes (Kasama et al., 1993). Monocytes then start to differentiate into two subtypes of macrophages (Figure 2). The pro-inflammatory macrophages, formerly termed M1 macrophages, secrete IL-1β, IL-6 and TNF-α inducing proliferation of myogenic cells. The anti-inflammatory macrophages, formerly termed M2 macrophages, release IL-4 and IGF-1 thereby promoting myogenic differentiation (Horsley et al., 2003; Dumont and Frenette, 2010; Saclier et al., 2013). Moreover, anti-inflammatory macrophages secrete different extracellular matrix (ECM) proteins which are important components of the MuSC niche and promote their self-renewal, among them Fibronectin and Collagen type VI (ColVI) (Gratchev et al., 2001; Schnoor et al., 2008; Bentzinger et al., 2013b; Urciuolo et al., 2013). Upon injury MuSCs get activated and enter the cell cycle (Bentzinger et al., 2010). They then become myogenic progenitor cells or fuse with the damaged myofibers after migration to the site of injury, a process which is controlled by signaling through Ephrins and Wnt7a (Stark et al., 2011; Bentzinger et al., 2014). Wnt signaling is one of the important signaling pathways in muscle regeneration. Wnt5a, Wnt5b, and Wnt7a are upregulated at early phases of regeneration while Wnt3a and Wnt7b expression increase at later phases (Polesskaya et al., 2003; Brack et al., 2008). While Wnt3a drives differentiation of MuSCs through activation of the canonical Wnt signaling pathway, Wnt7a promotes asymmetric MuSC division together with the ECM protein Fibronectin. Furthermore, Wnt7a induces migration of MuSCs and growth of myofibers through activation of different non-canonical Wnt pathways (Polesskaya et al., 2003; Brack et al., 2008; Otto et al., 2008; Le Grand et al., 2009; Bentzinger et al., 2014). Interestingly, Wnt7a always signals through Fzd7 in skeletal muscle activating different signaling pathways in the respective cell types, among them the planar cell polarity pathway and the AKT/mTOR pathway (von Maltzahn et al., 2012). A fine regulation of Wnt signaling is required for proper regeneration of skeletal muscle. For instance, increased canonical Wnt signaling during aging causes impaired regeneration of skeletal muscle and increased fibrosis (Brack et al., 2007). However, the anti-aging hormone soluble Klotho (sKlotho) is an antagonist of canonical Wnt signaling and important for maintaining MuSC functionality. This suggests that Klotho may be a naturally occurring inhibitor of increased canonical Wnt signaling in aged skeletal muscle and its availability could overcome over live time acquired changes in aged MuSCs (Ahrens et al., 2018). Furthermore, R-spondin plays a role in differentiation of myogenic progenitor cells during regeneration by positively regulating canonical Wnt signaling (Lacour et al., 2017). In addition to regulating Wnt activity, a temporal switch from Notch to canonical Wnt signaling is required for proper myogenic differentiation during regeneration (Brack et al., 2008). Here, Notch ligands control MuSC proliferation and differentiation (Conboy and Rando, 2002; Conboy et al., 2007; Brack et al., 2008; Mourikis et al., 2012). Especially the interplay between Notch and the transmembrane receptor Syndecan-3 (Sdc3) controls the maintenance of the MuSC pool and myofiber size after regeneration (Pisconti et al., 2010). Myogenic Regulatory Factors (MRFs) like Myf5, MyoD, Myogenin and Mrf4 facilitate myogenic differentiation of MuSCs allowing myogenic lineage progression required for regeneration of skeletal muscle (Braun et al., 1992; Rudnicki et al., 1992; Rudnicki et al., 1993; Singh and Dilworth, 2013). Myogenic progenitor cells become elongated and then fuse to form multinucleated myotubes expressing developmental myosin heavy chains (MHCs) (Bentzinger et al., 2010; Yin et al., 2013). In addition to the formation of new myofibers during regeneration reinnervation takes place, important for controlling MuSC behavior and maturation of myofibers (Vignaud et al., 2007; Forcina et al., 2020; Henze et al., 2024).

### Muscle stem cells and myogenic lineage progression in the adult

Regeneration of skeletal muscle is critically depending on MuSCs, a stem cell population residing underneath the basal lamina of myofibers first described by Alexander Mauro in 1961 (Mauro, 1961; Lepper et al., 2011; Murphy et al., 2011; Sambasivan et al., 2011; Schmidt et al., 2019) (Figure 1A). In adult skeletal muscle all MuSCs express the paired box transcription factor Pax7, which is essential for MuSC functionality, while subsets of them also express Pax3 or myogenic regulatory factor 5 (Myf5) (Relaix et al., 2005; Kuang et al., 2007; Lepper et al., 2011; Relaix and Zammit, 2012; Relaix et al., 2021). Although all MuSCs are expressing the canonical marker Pax7, the MuSC population is heterogeneous (Kuang et al., 2007; Mourikis et al., 2012; Chakkalakal et al., 2014). Under resting conditions MuSCs are quiescent but can be readily activated due to injury or other stimuli such as exercise (Fry et al., 2015; Schmidt et al., 2019).

After injury of skeletal muscle quiescent MuSCs become activated and then undergo myogenic lineage progression resulting in the expression of MyoD and Myf5. This causes their transformation into myogenic precursor cells (Chang and Rudnicki, 2014; Henze et al., 2020; von Maltzahn, 2021). However, MuSCs are capable of self-renewal thereby maintaining the MuSC pool and giving rise to myogenic progenitor cells required for regeneration of skeletal muscle (Blau et al., 2015). Myogenic differentiation is driven by the MRFs which comprise Myf5, MyoD, Myogenin and Mrf4, which control the process of elongation of myogenic progenitor cells into myocytes (Soleimani et al., 2012; Singh and Dilworth, 2013; Hernandez-Hernandez et al., 2017). Of note, fusion of myocytes into multinucleated myotubes depends on the expression of myomaker and myomerger (Millay et al., 2013; Leikina et al., 2018). The final step in regeneration of skeletal muscle is the maturation of myofibers which is coinciding with the migration of the centrally located nuclei into the periphery of myofibers (Forcina et al., 2020).

### Receptors in muscle stem cells

**FIGURE 3:**
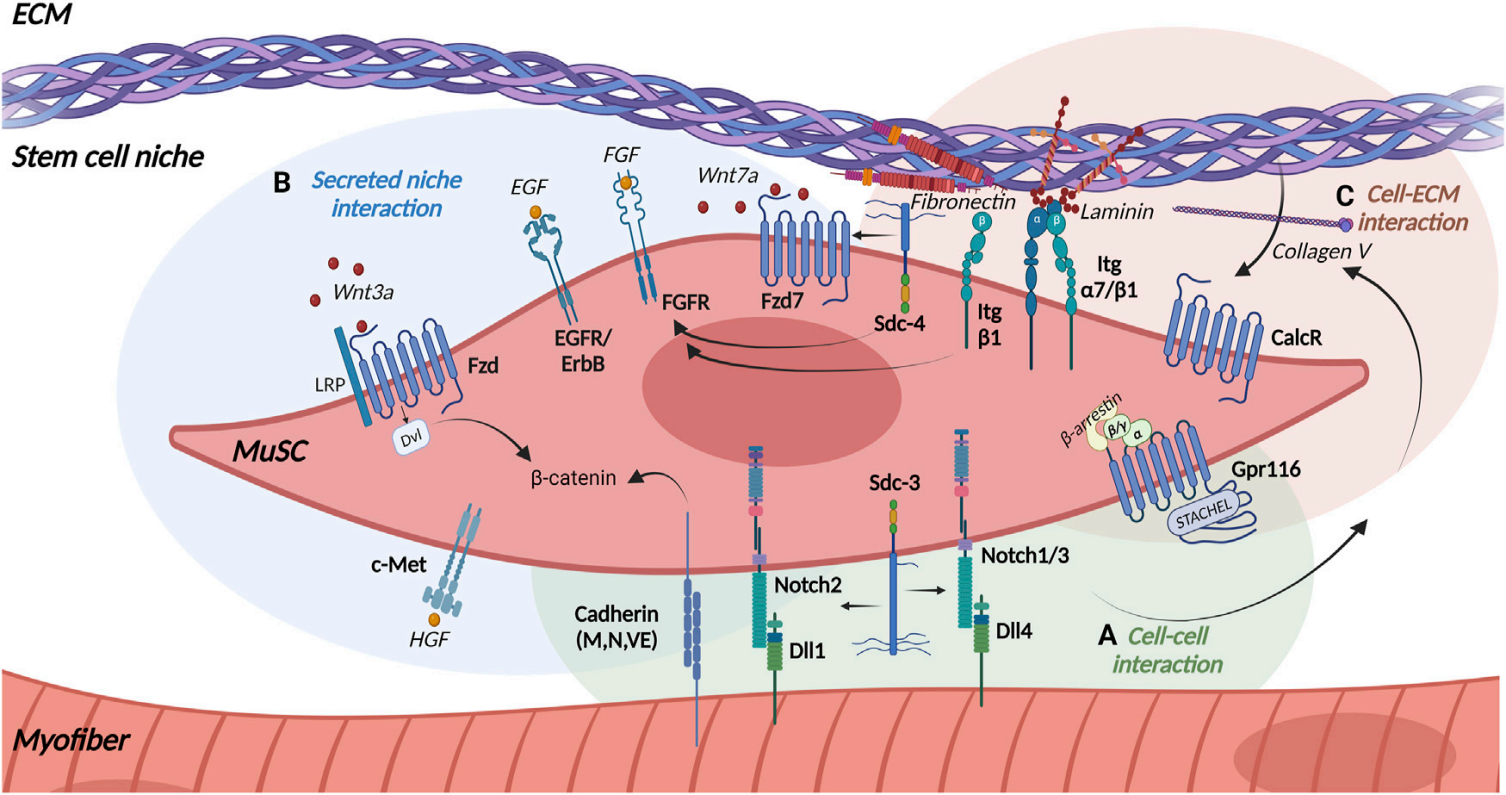
Receptors in MuSCs. MuSCs expresses various transmembrane receptors to interact with their local niche including **(A)** the myofiber (green background), **(B)** the stem cell niche (blue background), and **(C)** the extracellular matrix (ECM) (light brown background). Arrows indicate interaction partners. Abbreviations: Dvl, Dishevelled; LRP, Low density lipoprotein Receptor-related Protein; Wnt, Wingless-related integration site; Fzd, Frizzled receptor; Sdc, Syndecan; Itg, Integrin; Dll, Delta-like protein; Notch, Neurogenic locus notch homolog protein; EGF, Epidermal Growth Factor; EGFR, Epidermal Growth Factor Receptor; ErbB, Anti-apoptotic ErbB receptor; FGF, Fibroblast growth factor; FGFR, Fibroblast Growth Factor Receptor; HGF, Hepatocyte growth factor; c-Met, Mesenchymal epithelial transition factor; CalcR, Calcitonin receptor; Gpr116, adhesion G-protein-coupled receptor 116. Created with BioRender.com.

Proper regeneration of skeletal muscle requires an effective communication between the different cell types in skeletal muscle and MuSCs (Figure 2). MuSCs receive signals from the immediate niche and surrounding cells through a variety of transmembrane receptors. The interactions of signaling molecules with the transmembrane receptors in MuSCs activate signaling pathways which regulate their quiescence, activation and differentiation (Figure 3). Interactions of MuSCs with their surroundings can be divided into the following categories: direct cell-cell interactions (Figure 3A), signaling via secreted factors (Figure 3B) or cell-matrix interactions (Figure 3C) which we will discuss in detail in the following paragraphs.

## Direct cell-cell interactions

### Notch signaling

One of the main receptors in MuSCs controlling quiescence and differentiation are the Notch receptors. They are highly conserved single-pass transmembrane proteins with a large extracellular portion (Figure 3A). Mammals comprise four different Notch receptors (Notch 1–4) which are expressed on the cell surface of the signal-receiving cell. The Notch ligands Delta-like (Dll) −1, −4 and Jagged (Jag) −1, −2 are also transmembrane proteins located on the opposing signal-sending cell making a direct cell-cell communication a prerequisite for activation of the Notch signaling pathway. Activation of the Notch receptor by its ligands then leads to proteolytic cleavage of the receptor into a Notch extracellular domain (NECD) by Adam10 and into a Notch intracellular domain (NICD) by γ-secretases. The ligand remains bound to the extracellular part and is endocytosed by the signal-sending cell, while the cytosolic part migrates to the nucleus and binds to the transcription factor Recombination Signal Binding Protein for Immunoglobulin Kappa J Region (RBPJ) regulating the expression of Notch target genes (Vasyutina et al., 2007a; Kopan and Ilagan, 2009; Gioftsidi et al., 2022).

Notch signaling controls asymmetric division and quiescence of MuSCs. Importantly, high levels of Notch keep MuSCs in a quiescent state (Bjornson et al., 2012; Wen et al., 2012). The essential role of Notch signaling in maintaining MuSC quiescence was further supported by the finding that loss of Notch 1 and Notch 2 receptor in murine MuSCs results in break of quiescence (Fujimaki et al., 2018). Of note, expression of RBPJ, a downstream factor of Notch signaling, is a prerequisite for maintenance of MuSC quiescence (Bjornson et al., 2012; Mourikis et al., 2012). The genetic loss of RPBJ induces a break of quiescence and leads to spontaneous activation and premature differentiation of MuSCs (Bjornson et al., 2012; Bentzinger et al., 2013a). RBPJ and the Notch ligand Dll1 play an essential role in the maintenance of muscle progenitor cells (Vasyutina et al., 2007a), e.g. mutations in RBPJ and Dll1 lead to extensive and uncontrolled differentiation of progenitor cells resulting in an increased population of differentiated myogenic cells expressing MyoD and Myogenin and a reduced number of progenitors expressing Lbx1 and Pax3 (Vasyutina et al., 2007b; Schuster-Gossler et al., 2007). This uncontrolled myogenic differentiation leads to the depletion of the progenitor cell pool, resulting in insufficient muscle growth during development and severe muscle hypotrophy (Vasyutina et al., 2007a; Vasyutina et al., 2007b; Brohl et al., 2012).

While the Notch 2/Dll1 signaling pair was identified as the mediator of MuSC self-renewal (Yartseva et al., 2020), Dll1 also controls the differentiation of early myoblasts and the maintenance of myogenic progenitor cells in mouse embryos (Schuster-Gossler et al., 2007). In addition to its role in regulating MuSC functionality in the adult, Notch signaling plays an important role in embryonic and postnatal myogenesis controlling processes such as maintenance of the quiescent state, regulation of self-renewal and differentiation (Bjornson et al., 2012).

Furthermore, Notch signaling controls the interaction of MuSCs with their immediate niche, e.g., Notch1/RBPJ regulates the expression of the ECM molecule ColV, which promotes quiescence of MuSCs by binding to the Calcitonin receptor (CalcR) in an autocrine manner (Baghdadi et al., 2018) (Figures 3A, B). Notch also interacts with the single-pass transmembrane proteoglycan Sdc3 to regulate maintenance of the MuSC pool as well as self-renewal and reversible quiescence of MuSCs (Pisconti et al., 2010) (Figure 3). Syndecans interact with ECM proteins (e.g., Collagens, Laminins, Fibronectin) and growth factors (e.g., FGF-2, HGF, EGF, VEGF) via their ectodomain and with intracellular signaling molecules and cytoskeletal proteins through their intracellular domain (Leonova and Galzitskaya, 2013; Gondelaud and Ricard-Blum, 2019). Sdc3, along with Notch, is expressed in MuSCs and regulates their maintenance, proliferation and differentiation emphasizing the connection of the different signaling pathways (Fuentealba et al., 1999). Furthermore, Sdc3 controls myofiber size after regeneration and can be used as a membranous molecular marker to identify MuSCs next to Sdc4 (Pisconti et al., 2010; Wang et al., 2014).

### Cadherins

Quiescence of MuSCs is controlled by Notch signaling as well as through signaling via cadherins. Cadherins are single pass transmembrane glycoproteins, which mediate calcium-dependent cell-cell adhesion (Ivanov et al., 2001). Cadherins facilitate the direct binding of MuSCs to myofibers (Figure 3A). Three different cadherins are expressed by MuSCs and adult myofibers: M-, N- and VE-Cadherin. However, not all Cadherins appear to play similar roles in skeletal muscle (Kann et al., 2021). M-cadherin was found in quiescent and activated MuSCs and is one of the molecular markers of MuSCs (Wang et al., 2014). M- and N-cadherin regulate MuSC quiescence through the canonical Wnt/β-catenin signaling (Goel et al., 2017). In the absence of injury, removal of N-cadherin from adult MuSCs induces a break in quiescence, which can be enhanced by additional removal of M-cadherin. Removal of N-cadherin alone from MuSCs does not lead to an exit from the niche or loss of cell polarity, suggesting that the function of N-cadherin is rather related to maintenance of MuSC quiescence (Goel et al., 2017). Under homeostatic conditions, expression of M-cadherin in MuSCs mediates their adhesion to myofibers (Marti et al., 2013). Furthermore, M-cadherin is crucial for activation of cell division, e.g., *in vitro* treatment of MuSCs with M-cadherin stimulates cell division, whereas incubation with M-cadherin blocking antibodies reduces cell divisions (Marti et al., 2013).

### Gpr116

Another important regulator of MuSC quiescence is the adhesion G-protein-coupled receptor Gpr116, which belongs to the G-protein-coupled receptor (GPCR) superfamily. GPCRs are seven-pass-transmembrane receptors which are stimulated by extracellular ligands leading to the dissociation of the heterotrimeric G-protein (Gα, Gβ, Gγ) resulting in the activation of the respective intrinsic signaling cascades. Nevertheless, adhesion GPCRs have several atypical characteristics, including an exceptionally long extracellular N-terminus, which contains adhesion domains and a highly conserved region for autoproteolytic cleavage (Bassilana et al., 2019). Adhesion GPCRs like Gpr116 carry an agonistic sequence within the autoproteolysis-inducing (GAIN) domain. Short peptides derived from this region, called Stachel sequence*,* serve as a tethered agonist and can activate the respective receptor and initiate the respective signaling cascade (Stoveken et al., 2015; Demberg et al., 2017). Sénéchal and colleagues recently showed that adhesion GPCR Gpr116 is present at high levels in quiescent MuSCs being essential for long-term maintenance of the MuSC pool regarding quiescence and self-renewal capacity (Figure 3C). Of note, stimulation of MuSCs with the Gpr116 *Stachel* peptide prevents activation and differentiation of MuSCs. This stimulation also leads to a strong association with β-arrestins and increases the nuclear localization of β-arrestin 1, where it interacts with the cAMP response element binding protein (CREB) to regulate gene expression (Senechal et al., 2022). Furthermore, expression of Gpr116 is rapidly downregulated in activated MuSCs. MuSCs lacking Gpr116 are incapable of maintaining quiescence by showing progressive depletion over time and impaired self-renewal underscoring the importance of Gpr116 for maintenance of MuSC quiescence (Senechal et al., 2022).

## Interaction with secreted niche factors

### Wnt signaling

In addition to direct cell-cell-interactions controlling mainly MuSC quiescence, MuSC functionality is regulated by secreted niche factors, e. g., Wnt signaling regulating divisions of MuSCs. Wnt signaling through Frizzled (Fzd) receptors plays an important role in asymmetric division and migration of MuSCs. Fzd receptors are seven-pass transmembrane proteins with a large extracellular cysteine-rich domain (CRD), which is involved in ligand binding (Nusse, 2008; Sethi and Vidal-Puig, 2010; Clevers and Nusse, 2012). Fzd receptors are activated by different Wnt proteins, a large family of secreted glycoproteins, related to the wingless gene in *Drosophila* (Sethi and Vidal-Puig, 2010; Willert and Nusse, 2012). In mammals, the Wnt family comprises 19 members, with high amino acid sequence identities but distinct signaling properties resulting in multiple intracellular responses (Nusse, 2008).

The canonical Wnt signaling pathway, also known as Wnt/β-catenin pathway, requires the transmembrane low density lipoprotein receptor-related protein (LRP) as a co-receptor as well as the transcriptional activity of β-catenin (Nusse, 2012). β-catenin forms a degradation complex with axin, adenomatous polyposis coli (APC) and glycogen synthase kinase-3 beta (GSK-3β). In the absence of Wnt, β-catenin is phosphorylated within the degradation complex, leading to its own degradation (Katoh and Katoh, 2007). Binding of Wnt ligands to their respective Fzd receptors causes the activation of heterotrimeric G-proteins and the cytoplasmic phosphoprotein Dishevelled (Dvl). This results in a phosphorylation-dependent recruitment of axin to the Fzd co-receptor LRP and inactivation of the β-catenin degradation complex, followed by the accumulation and stabilization of β-catenin in the cytoplasm and its translocation into the nucleus. Here, β-catenin binds to the transcription factors T-cell factor (TCF) and lymphoid enhancer factor (LEF) and acts as a transcriptional coactivator inducing Wnt/β-catenin target genes (Abu-Elmagd et al., 2010; Grumolato et al., 2010). In adult skeletal muscle canonical Wnt signaling is mainly mediated through the Fzd ligand Wnt3a which drives differentiation of MuSCs (Otto et al., 2008; von Maltzahn et al., 2012) (Figure 3B). Upon activation of MuSCs canonical Wnt signaling increases and antagonizes the effects of Notch signaling. The temporal switch from Notch to Wnt signaling is essential for normal myogenesis regarding differentiation and progression of myogenic commitment (Brack et al., 2008). Additionally, maintaining a balanced and proper canonical Wnt signaling is crucial for successful regeneration. It has been demonstrated that R-spondin, a modulator of canonical Wnt signaling, plays an important role in differentiation of myogenic progenitor cells during regeneration (Lacour et al., 2017). Furthermore, it was shown that conditional activation or disruption of β-catenin in adult MuSCs also impairs regeneration of skeletal muscle (Rudolf et al., 2016).

In contrast to the canonical pathway, non-canonical Wnt ligands activate several non-canonical pathways in MuSCs and myofibers, such as the planar cell polarity, the PI3K/AKT/mTOR and the Wnt/Calcium pathway (von Maltzahn et al., 2011; von Maltzahn et al., 2013a; von Maltzahn et al., 2013b). Of note, all ligands signal through Fzd receptors independently of β-catenin and LRP (Nusse, 2012; von Maltzahn et al., 2012). In skeletal muscle, Wnt7a and its receptor Fzd7 mediate non-canonical Wnt signaling thereby regulating regeneration and growth of skeletal muscle (Bentzinger et al., 2014; Bentzinger et al., 2013b; Le Grand et al., 2009; Schmidt et al., 2022; von Maltzahn et al., 2011; von Maltzahn et al., 2013b) (Figure 3B). Wnt7a signaling specifically promotes symmetric satellite stem cell divisions, a subpopulation of MuSCs, via the formation of a coreceptor complex with the ECM glycoprotein Fibronectin and the receptor Sdc4 (Le Grand et al., 2009; Bentzinger et al., 2013b) (Figure 3B). Another Wnt family protein, Wnt4, is released by myofibers and controls MuSC quiescence by activating the Rho GTPase and repressing the Yes-associated protein (YAP) via a non-canonical Wnt pathway (Eliazer et al., 2019).

### FGF, EGF, and HGF signaling

While Wnt signaling mainly regulates MuSC divisions, FGF signaling preferentially controls proliferation of MuSCs. Fibroblast growth factor receptors (FGFRs) are receptor tyrosine kinases (RTKs) comprising the four homologous members FGFR1-4. Like all common RTKs, they contain an intracellular tyrosine kinase domain and a large extracellular ligand-binding domain, which binds fibroblast growth factors (FGFs) as their native ligands. FGFR signaling is involved in various physiological processes like proliferation, differentiation, cell migration and survival (Dai et al., 2019).

The expression of all four FGF receptors was shown in myofiber cultures and in MuSCs (Kastner et al., 2000). The fibroblast growth factors FGF-2 and FGF-6 regulate MuSC function via various signaling pathways including ERK MAPK, p38α/β-MAPK, PI3 kinase, PLCγ or STAT signaling (Pawlikowski et al., 2017). FGF-2 and FGF-6 promote proliferation of MuSCs and inhibit their differentiation in mice (Bentzinger et al., 2010; Pawlikowski et al., 2017). In rat myofiber cultures, FGF-1, FGF-4 and FGF-6 enhance proliferation of MuSCs similar to FGF-2 in mice (Kastner et al., 2000). FGF-6 is present at high concentrations in isolated myofibers, suggesting that the myofiber is the main source of FGF-6 *in vivo* (Kastner et al., 2000). The unique localization of FGF-6 and FGFR4 may have a specific function during myogenesis (Kastner et al., 2000). However, presumably FGF-6 has a dual role during myoblast proliferation, migration and muscle differentiation, hypertrophy and regeneration which is depending on the activation of distinct signaling pathways that recruit either FGFR1 or FGFR4 receptors in a dose-dependent manner (Armand et al., 2006). Proper FGF signaling in MuSCs requires the interaction with Sdc4, β1-Integrin and Fibronectin (Pawlikowski et al., 2017) (Figure 3B). Alteration or reduction of levels of either β1-Integrin, Fibronectin or Sdc4 modulates FGF signaling in MuSCs and affects their behavior (Pawlikowski et al., 2017). For example, an abnormal localization of β1-Integrin during aging leads to a diminished FGF-2 response, resulting in aberrant ERK signaling controlling activation of MuSCs (Rozo et al., 2016).

In addition to FGF receptors, MuSCs express another class of RTK receptors, the anti-apoptotic ErbB receptors which comprise four members: the epidermal growth factor (EGF) receptor (also known as ErbB1), ErbB2, ErbB3 and ErbB4. They are single-pass transmembrane proteins with an extracellular ligand-binding domain for EGF-related growth factors, and a cytoplasmic protein tyrosine kinase domain being able to form homo- and heterodimers (Olayioye et al., 2000) (Figure 3B). Golding et al. demonstrated in 2007 that MuSCs do not express any ErbB receptors in the quiescent state. However, within 6 h of activation ErbB1, ErbB2 and ErbB3 are expressed, while ErbB4 is activated in the first 24 h of activation. Furthermore, Golding and colleagues show that ErbB2 signaling plays a role in preventing apoptosis thereby preserving MuSCs during the critical phase of stem cell activation (Golding et al., 2007).

Receptors can be also used as molecular surface markers to identify MuSCs, among them c-Met and CXCR4 (Figure 3B). Mesenchymal epithelial transition factor (c-Met) is a single-pass, disulfide-linked α/β-heterodimer of the RTK family with high affinity to hepatocyte growth factors (HGF). Ligand/receptor interaction activates different signaling pathways, which are involved in proliferation, motility, migration, invasion and evasion of apoptosis (Organ and Tsao, 2011). c-Met is one of the molecular markers of MuSCs and required for regeneration of skeletal muscle (Webster and Fan, 2013; Wang et al., 2014; Lahmann et al., 2021). The study by Lahmann et al. (2021) showed that c-Met and *C-X-C* chemokine receptor type 4 (CXCR4) signaling cooperate during muscle regeneration. CXCR4 is a GPCR of the chemokine family, which is activated by the chemokine CXCL12 (Sdf-1α) and stimulates proliferation and migration of MuSCs (Vasyutina et al., 2005; Griffin et al., 2010). Consequently, MuSCs deficient of c-Met and CXCR4 are susceptible to apoptosis, while c-Met and CXCR4 signaling protects MuSCs from TNF-α-induced apoptosis (Lahmann et al., 2021).

## Cell-matrix interactions

### Integrin signaling

MuSCs are embedded in their niche. ECM molecules make up a large portion of the MuSC niche and regulate MuSC functionality. Here, Integrin receptors (Itg) are responsible for cell-matrix and cell-cell interactions. They function as extracellular receptors for ECM ligands such as Fibronectin, Laminin, Collagens or Vitronectin and thus form the structural and functional link between the ECM and intracellular cytoskeletal proteins (Figure 3C). Integrins consist of non-covalently bound α- and β-subunits. In the resting state they present in an inactive conformation, while binding of chemokines and growth factors results in their activation and binding of intracellular molecules such as Paxillin, Talin and Kindlin to the β-subunit thereby allowing binding to ECM components. This binding promotes the recruitment of signaling molecules such as Integrin Linked Tyrosine (ILK), Focal Adhesion Kinases (FAK) and modulation of signaling pathways such as AKT, ERK, Rho-GTPases and mTOR (Hynes, 2002; Takada et al., 2007; Campbell and Humphries, 2011; Taylor et al., 2022).

The heterodimer α7/β1-Integrin can be mainly found in skeletal muscle and has a high affinity for Laminin (Kramer et al., 1991; Loreti and Sacco, 2022) (Figure 3C). Quiescent MuSCs express high levels of α7- and β1-Integrin, which makes them good molecular markers of MuSCs (Blanco-Bose et al., 2001; Wang et al., 2014). Of note, β1-Integrin is involved in the maintenance of MuSC homeostasis as well as the expansion and self-renewal of MuSCs during regeneration. Moreover, β1-Integrin interacts with FGF-2 thereby controlling MuSC proliferation and self-renewal while β3-Integrin regulates differentiation of MuSCs in regenerating muscle (Liu et al., 2011; Rozo et al., 2016). Integrins also play an important role in the interaction of MuSCs with their immediate niche, MuSCs adhere to the ECM molecule Fibronectin via α4/β1-, α4/β7-and α5/β1-Integrins or to Laminin via α6/β1-Integrin (Figure 3C), interactions which are especially important during myogenesis (Taylor et al., 2022). Here, Fibronectin mediates the peripheral nuclear positioning through binding to α5-Integrin, a process depending on activation of FAK and the tyrosine kinase Src (Roman et al., 2018).

### Signaling through the calcitonin receptor

While the ECM molecules Fibronectin and Laminin mainly interact with Integrins, ColIV binding to the Calcitonin receptor (CalcR) regulates quiescence of MuSCs (Baghdadi et al., 2018) (Figure 3C). The CalcR is another member of the GPCRs which regulates quiescence of MuSCs, similar to Gpr116. Binding of the peptide hormone Calcitonin to the CalcR causes its activation resulting in the activation of multiple signaling pathways through its interaction with different G-protein family members (Gs and Gq) involved in maintaining calcium homeostasis (Masi and Brandi, 2007). In addition to regulating quiescence by signaling via the CalcR-protein kinase A (PKA)-Yes-associated protein 1 (Yap1) axis, CalcR is a molecular marker of MuSCs (Wang et al., 2014; Yamaguchi et al., 2015; Zhang et al., 2019; Zhang L. et al., 2021). MuSCs are retained in a quiescent state by the Notch-ColV-CalcR signaling pathway (Baghdadi et al., 2018). Here, ColV is produced as a result of Notch signaling and acts as a ligand for the CalcR. ColV production is reduced upon MuSC activation and inhibition of ColV synthesis leads to their activation and differentiation (Baghdadi et al., 2018).

### The MuSC niche and its remodeling after injury

Receptors in MuSCs are connecting MuSCs to the local environment, also known as the MuSC niche. The niche plays a prominent role in regulating quiescence and activation of MuSCs, myogenic differentiation and thereby regeneration of skeletal muscle. For instance, quiescence of MuSCs is regulated through the tight expression of multiple transcription factors in MuSCs. Binding of ECM components from the MuSC niche by receptors in MuSCs controls their expression and thereby the state of quiescence (Chang and Rudnicki, 2014). The ECM, a complex network of proteins and carbohydrates, provides structural support to MuSCs. Its composition is tightly connected to the age of an individual and state of regeneration regulating MuSC functionality. The most prominent components of the ECM in the MuSC niche include Collagens, Laminins, Vitronectin, Fibronectin and other glycoproteins as well as adhesion molecules such as M-Cadherin and CD34 (Casaroli Marano and Vilaro, 1994; Beauchamp et al., 2000). Post-translational modifications (PTMs) are essential for the proper functionality of ECM molecules and play an important role in regulating cellular behavior (Hu et al., 2022). These PTMs can occur at various stages of ECM protein synthesis, secretion or degradation and include mostly phosphorylation, glycosylation, acetylation and ubiquitination (Yuan and Ye, 2021).

The MuSC niche is severely remodeled during regeneration of skeletal muscle including a change in the composition of cell types in the immediate MuSC niche. Here, the interplay between MuSCs and various muscle resident cell types such as fibroblasts, immune cells (including macrophages, eosinophils and neutrophils) and FAPs affects and controls proper regeneration of skeletal muscle (Abou-Khalil et al., 2010; Lander et al., 2012). During regeneration dynamic remodeling of the ECM takes place, which is driven by changes in expression and thereby secretion of ECM molecules by MuSCs and other cell types in regenerating muscle. This remodeling causes alterations in MuSC behavior required for regeneration. For instance, MuSCs become activated through upregulation of Fibronectin expression and consequently activation of the respective receptors (Bentzinger et al., 2010; Shirakawa et al., 2022). Of note, activated mast cells create a pro-inflammatory environment after injury through secretion of cytokines, tryptase and TNF-α, which in turn is responsible for the downregulation of the expression of Pax7 (Palacios et al., 2010). Afterwards, monocytes differentiate into macrophages (pro-inflammatory and anti-inflammatory) which stimulate the early and late phases of myogenic processes by secretion of ECM components including Fibronectin and ColVI, respectively (Bentzinger et al., 2014; Mashinchian et al., 2018). In addition to MuSCs and immune cells, FAPs get activated after injury and rapidly increase in number (Sastourne-Arrey et al., 2023). They contribute to myogenic differentiation by secretion of the cytokine interleukin (IL)-6. However, eosinophils secrete additional cytokines such as IL-4 or IL-3, which are responsible for blocking the adipogenic differentiation of FAPs (Bentzinger et al., 2013a). Moreover, endothelial cells secrete a variety of antiapoptotic factors (e.g., VEGF) which stimulate the proliferation of MuSCs during regeneration (Frey et al., 2012).

### Alterations in regeneration of skeletal muscle in age and disease

As outlined above, regeneration of skeletal muscle is a highly orchestrated process in which each step is tightly controlled. During aging as well as in different disease states this precise control is out of balance resulting in impairments or delays of regeneration.

**FIGURE 4:**
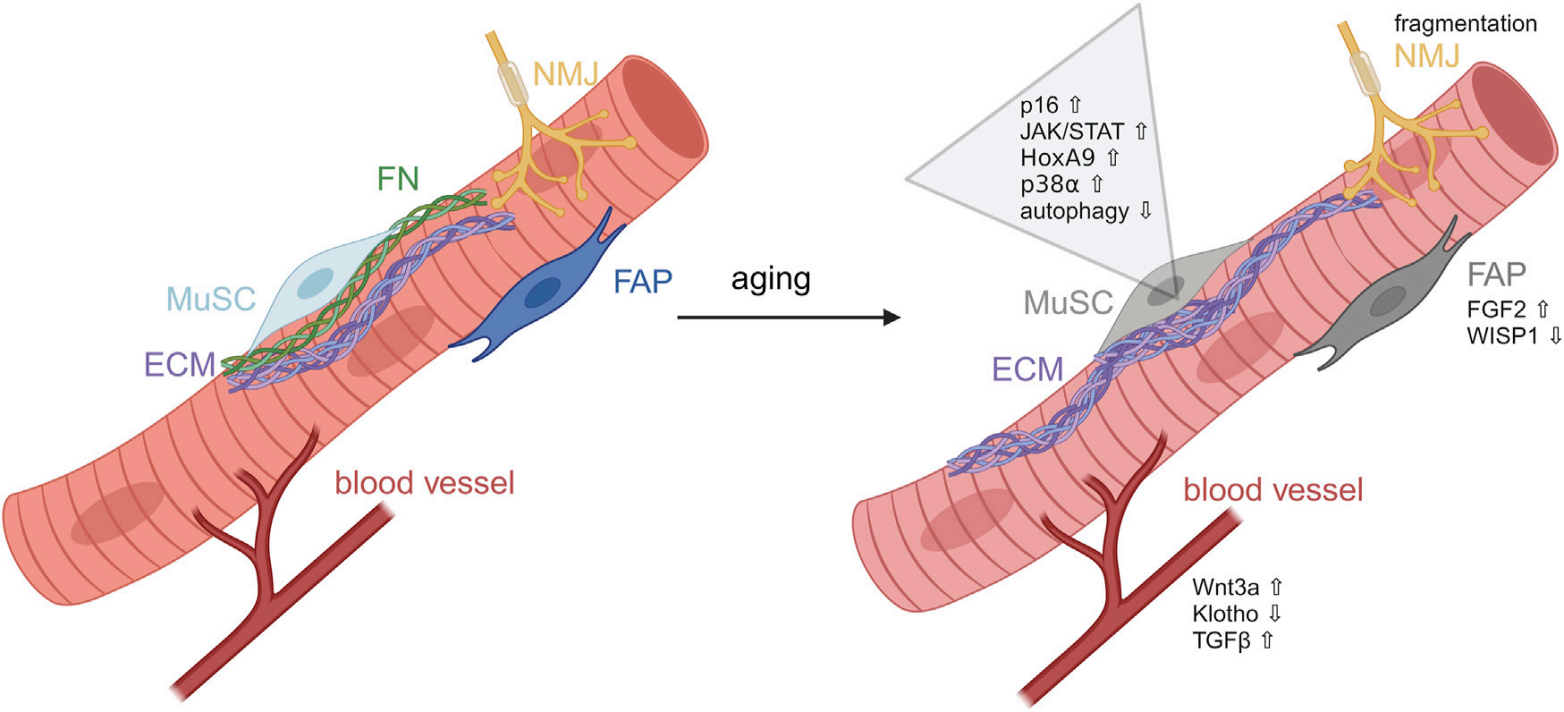
Alterations in MuSCs during aging. Induction of developmental pathways during aging impairing MuSCs functionality. MuSC, muscle stem cell; ECM, extracellular matrix; FN, fibronectin; FAP, fibro-adipogenic progenitor; NMJ, neuromuscular junction. Figure was modified from Henze et al. (2020).

Aging is characterized by a decline of organ function and integrity, accompanied by a decrease of regenerative capacity and an increase in vulnerability. The reduced ability of tissues to regenerate is mainly caused by stem cell exhaustion and deterioration (Kirkwood, 2005; Sousa-Victor et al., 2014; Lopez-Otin et al., 2023). Aging of skeletal muscle is marked by the gradual loss of muscle mass, strength and overall impaired physical performance, also called sarcopenia (Cruz-Jentoft et al., 2019). Additionally, muscle tissue is often replaced by adipose tissue (Rahemi et al., 2015; Yoshiko et al., 2017). During aging MuSCs switch to an irreversible cell cycle arrest and show increased levels of H3K27me3, which is associated with transcriptional repression (Liu et al., 2013; Sousa-Victor et al., 2014; Sousa-Victor et al., 2018) (Figure 4). Furthermore, functionality of MuSCs is impaired through the aberrant induction of developmental pathways caused by permissive chromatin states (Figure 4). For example, expression of Hoxa9 is induced and activates pathways such as JAK/STAT signaling limiting MuSC function (Schworer et al., 2016). Also, p38α/β-MAPK signaling displays aberrant upregulation in aged MuSCs inhibiting their self-renewal and thus regenerative potential (Cosgrove et al., 2014). Upregulation of developmentally important signaling pathways such as canonical Wnt signaling, JAK/STAT signaling and downregulation of Notch signaling further diminishes MuSCs functionality and drives them into a fibrogenic fate (Brack et al., 2007; Carlson et al., 2009; Price et al., 2014; Tierney et al., 2014) (Figure 4). In MuSCs from geriatric mice epigenetic p16INK4a depression is lost driving MuSCs into an irreversible pre-senescent state (Sousa-Victor et al., 2014; Schworer et al., 2016). An additional driver for loss of stem cell functionality with increasing age is their reduced autophagic activity leading to an accumulation of damaged mitochondria and increased ROS levels (Garcia-Prat et al., 2016). In addition to intrinsic changes in MuSCs, systemic factors show alterations during aging, e.g., serum levels of TGF-β1 are increased in elderly humans and mice which stimulates the expansion of tissue-resident fibroblasts and inhibits the myogenic differentiation of MuSCs, leading to a diminished regenerative capacity of aged muscle (Carlson et al., 2009). Furthermore, reduced levels of the well-known anti-aging hormone Klotho lead to a perturbed number and functionality of MuSCs resulting in a reduction of the regenerative capacity of skeletal muscle (Ahrens et al., 2018).

As outlined above, MuSCs are also directly affected by their local environment. Here, the ECM shows the biggest alterations during aging (Birch, 2018). With increasing age, systemic cytokine levels are altered and shift towards a low-grade chronic inflammation, a process also known as “inflammaging” (Franceschi et al., 2018). In skeletal muscle, this leads to the deregulation of ECM remodeling enzymes and their inhibitors, thereby increasing the amount of fibrotic tissue and impairing differentiation of myoblasts into myofibers (Blau et al., 2015). Additionally, the altered elasticity of fibrotic muscle tissue is likely to impair self-renewal of MuSCs (Urciuolo et al., 2013). Furthermore, it was shown that the direct interactions between MuSCs and the myofiber are controlling MuSC behavior (Bischoff, 1990). The exact mechanism for this interaction is not known but FGF-2 is increasingly secreted by aged myofibers and, at least in part, responsible for the age-related depletion of the MuSC pool (Chakkalakal et al., 2012). Increased FGF-2 levels hinder MuSCs to return to quiescence via constant activation of ERK signaling (Chakkalakal et al., 2012). In addition to alterations in the secretome of myofibers, the myofiber size seems to directly affect number and function of MuSCs, both of which are reduced during aging (Verdijk et al., 2007). Moreover, other muscle resident cell types change their functionality during aging, e.g., FAPs, which contribute to muscle homeostasis and regeneration, are displaying alterations during aging. For instance, it was shown that the matricellular protein Wisp1 is important to promote the expansion of MuSCs during regeneration. However, with increasing age Wisp1 secretion by FAPs is reduced, contributing to impaired MuSC functionality which then causes a reduced regenerative capacity. This is reminiscent of the observation that loss of Fibronectin expression in aged skeletal muscle impairs its regeneration (Lukjanenko et al., 2016; Lukjanenko et al., 2019).

With increasing age regeneration of skeletal muscle is reduced. However, other physiological states or diseases can also lead to an insufficient tissue restoration and/or maintenance of skeletal muscle. Among those are cancer cachexia, congestive heart failure, chronic obstructive pulmonary disease, chronic infectious diseases, neuromuscular diseases, chronic inflammatory diseases and acute critical illness. In those diseases functionality of MuSCs is affected through increased inflammation, oxidative stress, metabolic changes or unbalanced nutrition (Sharifi-Rad et al., 2020). Duchenne Muscular Dystrophy (DMD) pathology is one of the degenerative diseases affecting regeneration and maintenance of skeletal muscle. Here, the absence of the Dystrophin protein leads to sarcolemma instability and fragility. DMD is associated with extensive damage of myofibers upon contraction which cannot be rescued by newly regenerated myotubes (Ohlendieck et al., 1993; Grounds et al., 2008). Furthermore, divisions of MuSCs are affected in mdx mice, the mouse model of DMD (Dumont et al., 2015). Another example for muscle wasting diseases is myositis which affects proximal skeletal muscles and is clinically characterized by muscle weakness and a low level of muscle endurance (Lundberg et al., 2016). Here, an inflammatory cell infiltration, mainly composed of T-cells, macrophages and dendritic cells, occurs in skeletal muscle although the molecular mechanisms causing muscle wasting is not fully understood yet (Engel and Arahata, 1984; Greenberg et al., 2005). However, it was suggested that muscle weakness is caused by a loss of capillaries leading to tissue hypoxia and a loss of myofibers due to degeneration and necrosis of myofibers as a result of direct cytotoxic effects of T-cells (Emslie-Smith and Engel, 1990; Hohlfeld and Engel, 1991). Severe muscle wasting and loss of MuSC functionality is also occurring in cancer cachexia, the loss of muscle mass and functionality due to cancer. Here, Wnt7a was shown to effectively counteract muscle wasting through activation of the anabolic AKT/mTOR pathway as well as improve MuSC functionality (Schmidt et al., 2020). In addition to loss of muscle mass, cancer cachexia is associated with muscle damage which results in activation of MuSCs. Although MuSCs are activated, they fail to properly differentiate due to aberrant expression of Pax7 (He et al., 2013), a situation which shows similarities to rhabdomyosarcoma cells, a type of cancer cells thought to arise from myogenic precursor cells and which are also characterized by impaired myogenic differentiation.

### Rhabdomyosarcomas

Although skeletal muscle is a tissue which does not undergo extensive tissue replacement and proliferation in the adult–except after injury - myogenic cells undergo proliferation during development, the time when rhabdomyosarcomas (RMS) arise. Rhabdomyosarcomas are the most common soft-tissue sarcoma in children and resemble cells committed to the skeletal muscle lineage in embryonic and fetal stages of development (Wei et al., 2022). However, the cell of origin is not well characterized so far. Literature suggests that RMS tumors could be initiated by cells of myogenic origin or by cells of non-myogenic origin (Keller et al., 2004; Hatley et al., 2012; Blum et al., 2013; Drummond et al., 2018).

RMS can be divided in two main subtypes, the most common embryonal rhabdomyosarcoma [ERMS, (∼70%)] and the more aggressive alveolar rhabdomyosarcoma [ARMS, (∼20%)]. The remaining RMS cases are caused by pleomorphic and spindle cell/sclerosing RMS (Ognjanovic et al., 2009). Classification in the clinics is mainly done by morphological and cytological assessment of hematoxylin and eosin-stained histology sections (Asmar et al., 1994; Davicioni et al., 2009). RMS tumors tend to occur at three main anatomical regions of the human body including the head and neck regions, the genitourinary system and the extremities (Arndt and Crist, 1999; Ma et al., 2015). However, RMS tumors can arise also at other locations in the human body. Of note, in all types of RMS a deregulated myogenic differentiation program leads to continuous proliferation and impaired terminal myogenic differentiation (Skapek et al., 2019).

The genetic alterations in most ARMS cases (approximately 80%) are well understood, here a chromosomal translocation between the *PAX3* [t (2; 13) (q35; q14)] or *PAX7* [t (1; 13) (q36; q14)] and Forkhead box protein O1 (*FOXO1*) occurs. This results in fusion genes thereby generating oncogenic transcription factors consisting of the DNA binding domain of the PAX and the transactivation domain of FOXO1, PAX-FOXO1. A minority of ARMS cases (∼20%) lacks these translocations and shares clinical and biological features of ERMS (Parham and Barr, 2013). The presence of a PAX-FOXO1 fusion (fusion-positive/FP RMS cases) drives unfavorable outcomes in children and is recognized as an important prognostic factor (Hibbitts et al., 2019). Both PAX-FOXO1 fusion proteins show more transcriptional activity, are expressed at a higher level and are proteolytically more stable than their wild-type PAX counterparts (Davis and Barr, 1997; Bennicelli et al., 1999; Miller and Hollenbach, 2007). Thereby, they contribute to tumorigenicity through affecting growth, apoptosis, differentiation and cell migration. The enhanced expression of PAX3 or PAX7 in ARMS–here as a fusion protein–is reminiscent of MuSCs in cancer cachexia which are also displaying aberrant high levels of Pax7 expression (He et al., 2013). The aberrant expression of Pax7 or Pax3 might be one of the main drivers of impaired myogenic differentiation in ARMS as observed in MuSCs in cancer cachexia.

While ARMS tumors are classified as fusion-positive tumors ERMS tumors are fusion-negative and show a high variability in the genetic alterations causing cancer. Among those alterations are a loss of heterozygosity at chromosome 11p15.5, an increase in aneuploidy, mutations of *TP53*, *RAS* genes, *PIK3CA*, β-catenin and *FGFR4*, as well as *NF1*, *FBXW7* and *BCOR* affecting RTK-RAS-RAF-MAPK, PI3K-AKT-mTOR signaling, cell cycle progression, apoptosis and developmental pathways such as Wnt, Notch, SHH and Hippo among others (Scrable et al., 1989; Stratton et al., 1989; Taylor et al., 2009; Zibat et al., 2010; Annavarapu et al., 2013; Shern et al., 2014; Mohamed et al., 2015; Conti et al., 2016; Skapek et al., 2019). Interestingly, mutations in ERMS often affect signaling pathways and receptors which also control MuSC functionality (Figure 3). However, in the vast majority of ERMS tumors the transcriptional repressor TRPS1 displays an increased expression causing impaired myogenic differentiation (Huttner et al., 2023). Of note, reduction of aberrant TRPS1 levels in ERMS tumor cells permits myogenic differentiation (Huttner et al., 2023).

All RMS tumors are diagnosed by the expression of myogenic markers such as the myogenic regulatory proteins MYOD and MYOGENIN, MHCs, skeletal α-ACTIN, Creatine Kinase and DESMIN (Tonin et al., 1991; Dias et al., 2000; Sebire and Malone, 2003). Histologically ERMS resembles an undifferentiated and embryonal state, while ARMS tumors are characterized by a more widely expression of key myogenic regulatory factors responsible for terminal differentiation such as MYOD and MYOGENIN (DeMartino et al., 2023). RMS treatment involves a multimodal approach including surgical excision, chemotherapy and radiation therapy. The outcome of metastatic or recurrent RMS patients remains poor, but localized instances are curable (Malempati and Hawkins, 2012; Dantonello et al., 2013). In recent decades, chemotherapy regimens have steadily improved, but remain non-specific to the tumor and include the application of vincristine, actinomycin D combined with cyclophosphamide or ifosfamide. However, recent modifications of these standard regimens have shown improvements in the outcomes of patients with rhabdomyosarcoma (Chen et al., 2019; Miwa et al., 2020). Nevertheless, additional treatment options for RMS would be desirable, potentially through inducing myogenic differentiation in tumor cells.

## Conclusion

Skeletal muscle is the most abundant tissue of the human body, it is characterized by a high plasticity and ability to self-renew. Skeletal muscle supports mobility and body posture. Any kind of muscle impairments, such as disease, aging, injury, etc. has an impact on the general health and therefore quality of life. Regeneration of skeletal muscle is a highly orchestrated process involving the reception of signals from the niche through a variety of receptors located in the plasma membrane of MuSCs. A better understanding of the interplay of the different cell types and signaling pathways during regeneration of skeletal muscle is required, especially in age and disease. A focus on the secretome of the different cell types in skeletal muscle and how the secreted factors are affecting MuSC functionality might be a promising approach to the development of new therapies for improving regeneration of skeletal muscle.
